# Quasi-1D NbTe_4_ for Broadband Pulse Generation from 1.0 to 3.0 μm: Bridging the Near- and Mid-Infrared

**DOI:** 10.3390/nano15060424

**Published:** 2025-03-10

**Authors:** Zian Cai, Wenyao Zhang, Qi Kang, Hongfu Huang, Xin Xiang, Shunbin Lu, Qiao Wen

**Affiliations:** 1 State Key Laboratory of Radio Frequency Heterogeneous Integration, College of Physics and Optoelectronic Engineering, Shenzhen University, Shenzhen 518060, China; 2 Key Laboratory of Optoelectronic Devices and Systems of Ministry of Education and Guangdong Province, College of Physics and Optoelectronic Engineering, Shenzhen University, Shenzhen 518060, China; 3 College of Medical Engineering and Technology, Xinjiang Medical University, Urumqi 830017, China; 4 International Collaborative Laboratory of 2D Materials for Optoelectronic Science & Technology of Ministry of Education, Institute of Microscale Optoelectronics (IMO), Shenzhen University, Shenzhen 518060, China

**Keywords:** NbTe_4_ nanosheets, broadband saturable absorber, passively Q-switched, passively mode-locked

## Abstract

Quasi-one-dimensional (quasi-1D) transition metal chalcogenides (TMCs), a subclass of low-dimensional materials, have attracted significant attention due to their unique optical and electronic properties, making them promising candidates for nonlinear photonics. In this work, NbTe_4_, a quasi-1D transition metal tetrachalcogenide, was synthesized and employed for the first time as a broadband saturable absorber (SA) for pulsed laser applications. The nonlinear optical (NLO) properties of NbTe_4_ were systematically characterized at 1.0 μm, 2.0 μm, and 3.0 μm, revealing saturation intensities of 59.53 GW/cm^2^, 14 GW/cm^2^, and 6.8 MW/cm^2^, with corresponding modulation depths of 17.4%, 5.3%, and 21.5%. Utilizing NbTe_4_-SA, passively Q-switched (PQS) pulses were successfully generated in the 1.0 μm and 2.0 μm bands, achieving pulse durations of 86 ns and 2 μs, respectively. Furthermore, stable mode-locked operation was demonstrated in an Er-doped fluoride fiber laser at 3.0 μm, yielding a pulse duration of 19 ps. These results establish NbTe_4_ as a highly promising broadband SA material for next-generation ultrafast photonic devices and pave the way for the development of other quasi-1D materials in nonlinear optics.

## 1. Introduction

Low-dimensional materials have attracted considerable attention in recent academic research due to their strong confinement effects and tunable physical properties, especially for quantum dot (QD) materials, two-dimensional (2D) materials, and quasi-one-dimensional (quasi-1D) materials, which enable pronounced interactions between light and matter. Over the past few decades, extensive efforts have been devoted to exploring these low-dimensional materials and their potential applications in various fields, including optoelectronics, bioelectronics, laser technology, energy storage devices, and optical modulators. Among them, QDs exhibit strong quantum confinement effects, and their size-dependent optical properties often lead to inhomogeneous broadening, which can limit their stability in certain applications. Meanwhile, two-dimensional (2D) materials have been widely studied, encompassing graphene [1], black phosphorus [2], topology insulators (e.g., Bi_2_Te_3_) [3,4], indium sulfide (e.g., In_2_S_3_) [5], transition metal dichalcogenides (e.g., MoS_2_ and WS_2_) [6,7,8], IV-VI semiconductors (e.g., SnS_2_ and SnSe_2_) [9,10], and Mxenes [11,12,13,14]. These materials have been effectively employed as broadband saturable absorbers (SAs) in lasers, facilitating the generation of Q-switched and mode-locked pulses with remarkable nonlinear absorption properties, but their interaction with light is often constrained to the plane of the material, limiting their effective nonlinear coefficient.

In contrast to conventional 2D materials, quasi-one-dimensional (quasi-1D) van der Waals (vdW) materials exhibit strong covalent bonds along the 1D chain direction, while weak vdW interactions exist between adjacent chains, as shown in Appendix A.1. The presence of additional bonding between chains facilitates the self-organization of 1D chains into 2D sheets. Similar to other 2D structures, these sheets are stacked into three-dimensional (3D) bulk crystals via weak vdW forces. These unique structural characteristics endow quasi-1D vdW materials with a combination of 1D and 2D properties [15]. Consequently, these materials exhibit diverse and intriguing electronic characteristics, including tunable bandgaps, charge density waves (CDWs), and superconductivity (SC), as well as highly anisotropic optical, thermoelectric, and nonlinear optical properties. For instance, the unique quasi-1D structure of single-walled carbon nanotubes (SWCNTs) grants them exceptional mechanical, electrical, and thermal properties, while their nonlinear excitonic optical behavior has been extensively studied for SA applications [16]. In solar cell applications, Sb_2_S_3_ exhibits carrier mobility and conductivity along the [001] direction that is approximately two orders of magnitude higher than along the [100] and [010] orientations due to its quasi-1D crystal structure [17]. Similarly, Ca(BO_2_)_2_, which consists of well-aligned 1D anion chains of corner-connected planar sp^2^-hybridized BO_3_ groups, displays significant anisotropy in polarizability, leading to large birefringence [18]. Transition metal trichalcogenides (TMTCs), commonly referred to as MX_3_ compounds (M = Ti, Zr, Hf, V, Nb, and Ta; X = S, Se, and Te), are typical quasi-1D vdW materials. Among them, ZrTe_3_ and NbSe_3_ exhibit unique CDW transitions and superconductivity, making them promising candidates for radio frequency nanoelectronic devices, as well as for applications in information processing and quantum computing [14]. Furthermore, owing to their strong in-plane anisotropy, ZrS_3_ nanosheets enable broadband and polarized photodetection with high tunability [19].

In recent years, there has been growing interest in chalcogen-rich transition metal chalcogenides, particularly tetrachalcogenides MX_4_ (M = V, Nb, and Ta; X = S, Se, and Te) [20]. For example, VS_4_, which exhibits a unique 1D atomic-chain structure, has been identified as a promising electrode material for high-performance batteries [21,22,23]. Additionally, metallic transition metal tetrachalcogenides, such as MTe_4_ (M = V, Nb, and Ta), have attracted substantial attention as a platform for investigating fundamental physical properties due to their distinctive quasi-1D structures [24]. The emergence of superconductivity in quasi-1D TaTe_4_ under pressure, observed through high-pressure electrical transport measurements, suggests that increased charge carrier density plays a crucial role in this phenomenon [25]. Moreover, NbTe_4_, as a phase-change material, has demonstrated exceptional switching performance in memory cells, achieving rapid switching speeds (30 ns) and exhibiting a Set/Reset resistance window exceeding two orders of magnitude [26]. Additionally, NbTe_4_ undergoes successive CDW transitions and superconductivity at a temperature of 2.2 K under hydrostatic pressure [27]. These studies highlight the significant potential of MTe_4_ (M = V, Nb, and Ta) in various technological fields. However, their application as SAs in solid-state lasers remains largely unexplored. Investigating the nonlinear optical (NLO) properties of MTe_4_ and developing solid-state pulsed lasers based on these materials would be highly beneficial for advancing photonic technologies.

NbTe_4_, as a quasi-1D material, maintains a well-defined electronic structure along its chain-like configuration, ensuring more stable and predictable nonlinear optical responses. Moreover, NbTe_4_ benefits from its quasi-1D structure, which enhances light–matter interaction along its chain direction while retaining high anisotropy, making it suitable for polarization-sensitive applications and achieving broadband NLO properties through symmetry-breaking distortions. Meanwhile, NbTe_4_ has a combination of strong nonlinear response, high carrier mobility, and structural stability. We anticipate that NbTe_4_ will serve as a promising candidate for ultrafast optical modulators and nonlinear frequency converters.

In this study, we investigate the broadband nonlinear absorption behavior of NbTe_4_ nanosheets, synthesized using the ultrasound-assisted liquid-phase exfoliation (LPE) technique. The structural characteristics of the prepared NbTe_4_ nanosheets are examined using X-ray diffraction (XRD), atomic force microscopy (AFM), and high-resolution transmission electron microscopy (HRTEM). Their optical properties are analyzed through Raman spectroscopy and transmission spectra measurements. For the first time, we fabricated a novel SA based on NbTe_4_, demonstrating pronounced NLO properties at 1.0 μm, 2.0 μm, and 3.0 μm, with saturation intensities of 59.53 GW/cm^2^, 14 GW/cm^2^, and 6.8 MW/cm^2^ and modulation depths of 17.4%, 5.3%, and 21.5%, respectively. By incorporating the fabricated NbTe_4_-SA, we successfully demonstrate passively Q-switched (PQS) solid-state lasers at 1.0 μm and 2.0 μm, as well as a mode-locked Er-doped fluoride fiber laser at 3.0 μm, achieving pulse durations of 86 ns, 2 μs, and 19 ps, respectively. These findings present a new avenue for utilizing quasi-1D tetrachalcogenides in laser and photonics applications.

## 2. Materials and Methods

### 2.1. Synthesis of NbTe_4_ Powder

NbTe_4_ powder was synthesized using a high-temperature solid-state reaction while maintaining a stoichiometric ratio of the constituent elements. The synthesis process involved the following steps: (1) weighing and mixing Nb and Te powders in the appropriate stoichiometric ratio, followed by loading the mixture into a graphite crucible, which was then placed inside a silica tube; (2) evacuating the silica tube to a vacuum of 10^−3^ Pa and sealing it using a flame; (3) placing the sealed silica tube inside a muffle furnace; (4) gradually heating the tube to 800 °C at a rate of 20 °C h^−1^ and maintaining this temperature for 100 h, followed by slow cooling to room temperature. Following this procedure, polycrystalline NbTe_4_ powder was obtained.

### 2.2. Preparation of NbTe_4_ Nanosheets and NbTe_4_-Based Saturable Absorber

**Figure 1:**
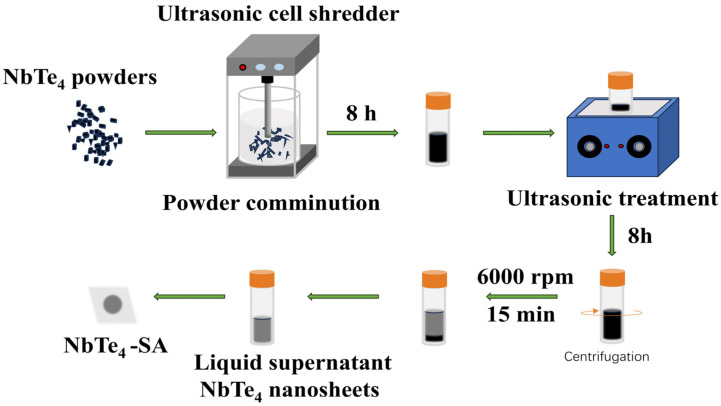
Preparation process of the NbTe_4_-SA.

As shown in Figure 1, to obtain a large quantity of few-layer NbTe_4_ nanosheets for the fabrication of a NbTe_4_-based saturable absorber (NbTe_4_-SA), the LPE method was employed. First, the synthesized NbTe_4_ powder was mechanically milled and subsequently dispersed in isopropyl alcohol (IPA). The dispersion was then subjected to ultrasonic treatment (200 W; 8 h), with careful control of sonication duration to prevent structural degradation due to overheating. Following this, the mixture was centrifuged at 6000 rpm for 15 min, and the supernatant, containing few-layer nanosheets, was extracted. The obtained nanosheets were uniformly deposited onto a 10 × 10 × 0.2 mm^3^ glass substrate and a gold mirror with a diameter of 25.4 mm. Finally, the sample was dried at 60 °C for 2 h in a vacuum oven to remove residual IPA, yielding the NbTe_4_-SA.

### 2.3. Nonlinear Optical Response of NbTe_4_-Based Saturable Absorber

**Figure 2:**
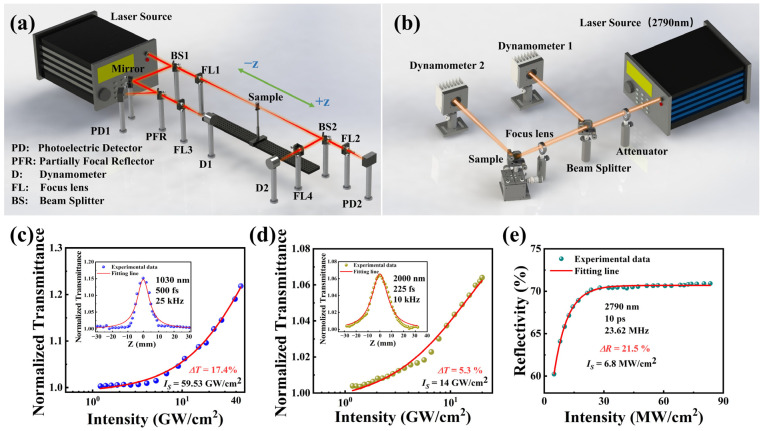
Nonlinear optical characterization of NbTe_4_. (**a**) Experimental setup of the OA Z-scan system. (**b**) Experimental device of the P-scan system. Relationship between the normalized transmittance and input laser intensity at (**c**) 1030 nm and (**d**) 2000 nm, respectively. (Inset: corresponding OA Z-scan curve.) (**e**) Saturable absorption cure at 2790 nm.

In this study, the nonlinear optical properties of NbTe_4_-SA were investigated using an open-aperture (OA) Z-scan measurement system and a P-scan measurement system. The nonlinear optical response of NbTe_4_-SA arises from its saturable absorption characteristics. For the principle of saturable absorption, please refer to Appendix A.2. The OA Z-scan system, depicted in Figure 2a, was employed for measurements at 1.0 μm and 2.0 μm. A femtosecond laser source with a central wavelength of 1030 nm was utilized, delivering pulses with a duration of 500 fs at a repetition rate of 25 kHz. For 2.0 μm, we used a femtosecond laser source with a wavelength of 2000 nm, a pulse width of 225 fs, and a repetition rate of 10 kHz. The laser output was split by a 50:50 beam splitter into two beams: the reflected beam served as the reference, while the transmitted beam was used for testing. The reference beam was further divided into two paths for waveform monitoring and power measurement. The test beam was focused onto the sample using a lens with a focal length of 150 mm. The transmitted laser pulse was subsequently measured using a power meter (D2) and a photodetector (PD2).

The NbTe_4_ sample was placed in a cuvette with a thickness of 1 mm. By adjusting the electric displacement platform along the *z*-axis, transmission pulse parameters under varying conditions were obtained, enabling the construction of the OA Z-scan curve [28]. Based on this curve, the relationship between transmittance and input laser intensity was derived. The nonlinear absorption fitting curve is described by the following equation [29]:

(1) $$T\left(I\right)=\left[1-∆T\times exp\left(-\frac{I}{I_{S}}\right)-\alpha_{ns}\right]/T_{0}$$

where $T\left(I\right)$ is the normalized transmittance, ∆T is the modulation depth, I is the input light intensity, $I_{S}$ is the saturable absorber intensity, $\alpha_{ns}$ is the unsaturated absorption intensity, and $T_{0}$ is the linear transmittance. As shown in Figure 2c,d, the corresponding modulation depth of 1.0 μm and 2.0 μm is 17.4% and 5.3%, respectively. The saturation intensity is 59.53 GW/cm^2^ at 1.0 μm and 14 GW/cm^2^ at 2.0 μm. A typical saturable absorption behavior was observed, where the transmittance increased with increasing incident light intensity before reaching saturation.

Due to the limitations of experimental conditions, we constructed a P-scan experimental setup to quantify the saturable absorption of NbTe_4_-SA at a wavelength of 3.0 μm. As illustrated in Figure 2b, a 3.0 μm semiconductor saturable absorber mirror (SESAM)-based passively mode-locked fiber laser, with a pulse duration of 10 ps, was employed to measure the reflectivity curve of NbTe_4_-SA under varying incident pulse fluxes. A detailed description of the setup is provided in Appendix B.1. The reflectivity curves corresponding to different peak pulse powers are presented in Figure 2e, and the experimental data were fitted using the following equation:

(2) $$R\left(I\right)=1-\Delta R\times \exp\left(-I/I_{sat}\right)-R_{ns}$$

where $R\left(I\right)$ represents the reflectivity, ∆R is the modulation depth, I denotes the input pulse energy, $I_{Sat}$ is the saturation energy, and $R_{ns}$ corresponds to the non-saturable loss. The calculated saturable power of NbTe_4_-SA was determined to be 6.8 MW/cm^2^, with a modulation depth of 21.5%.

The results demonstrate that NbTe_4_ nanosheets exhibit significant nonlinear absorption across the 1.0–3.0 μm range, highlighting their potential as broadband saturable absorbers for pulsed laser applications. A higher saturation intensity corresponds to greater saturable absorption loss, while modulation depth plays a crucial role in optimizing pulse duration. In passively Q-switched lasers, higher saturation intensity and modulation depth enhance energy storage, enabling high-energy, narrow-pulse outputs. However, increased pump power is required to reach saturation, which reduces conversion efficiency. In passively mode-locked lasers, the modulation capability of the saturable absorber should align with the laser structure. A lower saturation intensity facilitates mode-locked initiation at lower pump power, while sufficient modulation depth is essential for suppressing gain competition and noise perturbations, thereby ensuring stable self-starting mode-locked operation. Experimental results confirm that the 1.0 μm and 2.0 μm bands are well-suited for Q-switched operation, whereas the 3.0 μm band is more suitable for mode-locked operation, necessitating distinct resonator designs and output parameter optimization.

### 2.4. Laser Application of NbTe_4_-SA in Broadband Absorption

**Figure 3:**
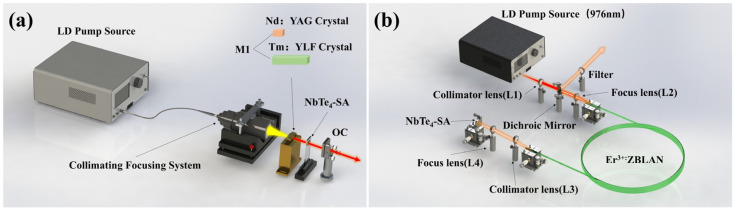
Experimental setups of the lasers based on NbTe_4_-SA. (**a**) PQS solid-state lasers operating at 1.0 μm and 2.0 μm. (**b**) Mode-locked Er-doped fluoride fiber laser at 3.0 μm.

To investigate the modulation performance of NbTe_4_-SA in a 1.0 μm laser system, a compact solid-state laser cavity was designed, achieving Q-switching operation by incorporating multiple layers of NbTe_4_ thin films into the cavity. The experimental setup is illustrated in Figure 3a. A Nd:YAG crystal (3 × 3 × 4 mm^3^; 1.2 at.%) cut along the [111] plane was used as the gain medium. A commercial 808 nm fiber-coupled diode laser (fiber core diameter: 400 μm; NA: 0.22) served as the pump source, delivering a maximum output power of 30 W. A 1:0.5 collimation focusing system was employed to precisely focus the pump beam onto the crystal with a spot diameter of 200 μm. The gain crystal, wrapped in indium foil, was mounted on a custom-designed water-cooled copper block, with the circulating water temperature maintained at 17 °C for efficient heat dissipation. The input mirror (M1) was coated with a dichroic film (HT@808 nm; HR@1064 nm; R = ∞) to optimize the pump light coupling, while the film at the opposite end (HR@808 nm; AR@1064 nm) enhanced absorption efficiency and minimized the influence of residual pump light on NbTe_4_-SA. The output coupler (OC) was a concave mirror with a 50 mm radius of curvature and 5% transmittance at 1064 nm. M1 and OC formed a plano-concave cavity with a length of 25 mm.

To evaluate the applicability of NbTe_4_-SA in 2.0 μm laser systems, a similar single-mirror flat-cavity configuration was developed, utilizing a Tm: YLF crystal (3 × 3 × 10 mm^3^, 3.0 at.%; A-cut) as the gain medium. The experimental setup for the 2.0 μm PQS laser is also depicted in Figure 3a. Further details regarding the experimental design are provided in Appendix B.2.

To examine the saturable absorption properties of NbTe_4_-SA at 3.0 μm, a linear fiber laser cavity was constructed to enable both Q-switching and mode-locking operations. The experimental configuration is shown in Figure 3b. A commercially available fiber-coupled laser diode with an emission wavelength of 976 nm was employed as the pump source, delivering a maximum output power of 60 W. An aspheric lens (L1) with an 18 mm focal length was positioned after the diode output to collimate the pump light. A dichroic mirror with 95% transmittance at 976 nm and >99.5% reflectivity at 2780 nm was placed behind L1 to separate the pump beam from the laser output. The gain medium consisted of a 4 m-long double-clad Er-doped fluoride fiber with a doping concentration of 70,000 ppm. The fiber core had a diameter of 15 μm and a numerical aperture (NA) of 0.12, while the circular inner cladding had a diameter of 260 μm. To couple the pump light into the inner cladding of the gain fiber, an anti-reflection-coated plano-convex lens (L2) with a 25 mm focal length was employed. The fiber end facing the pump beam was perpendicularly cleaved to provide a 4% Fresnel reflection, functioning as an output coupler, while the opposite end was cleaved at an 8° angle to suppress parasitic oscillations. The fiber ends were coated with a graphite film and mounted on a copper V-groove translation stage for efficient heat dissipation. To collimate and focus the output laser beam onto a gold reflector, a pair of plano-convex lenses (L3 and L4) with 25 mm focal lengths was used. The NbTe_4_-SA was deposited on the gold reflector to modulate the laser output. A long-pass filter was placed at the laser system output to block residual 976 nm pump light and background noise. Additional details regarding the instrumentation used in these laser experiments can be found in Appendix B.3 and Appendix B.4.

## 3. Results and Discussion

### 3.1. Characterization of NbTe_4_ Nanosheets

**Figure 4:**
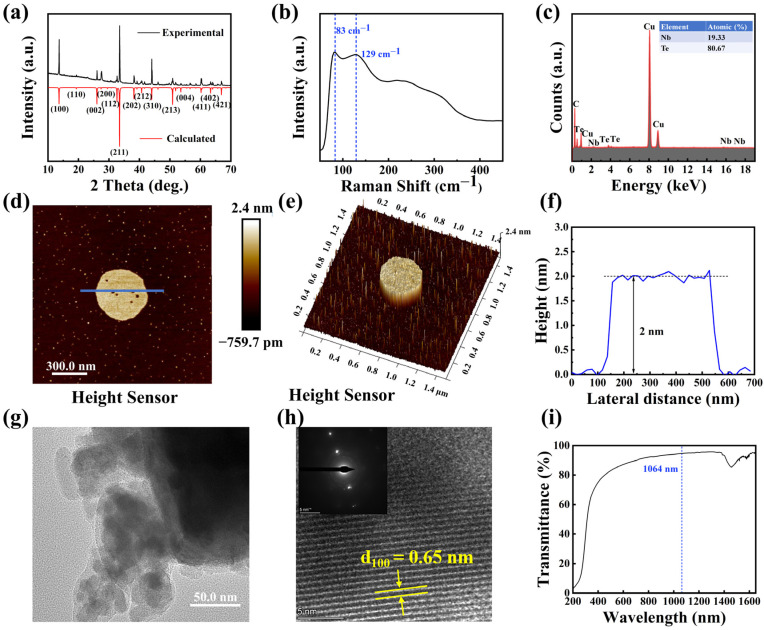
Characterization of the NbTe_4_. (**a**) Simulated and experimental XRD patterns of the NbTe_4_ polycrystalline powder. (**b**) Raman spectrum of NbTe_4_ nanosheets. (**c**) EDS spectrum. (**d**) AFM and (**e**) corresponding 3D morphology of NbTe_4_ nanosheets. (**f**) Thickness distribution of NbTe_4_ nanosheets. (**g**,**h**) HRTEM images of exfoliated NbTe_4_ nanosheets at scales of 50 nm and 5 nm, respectively. (**i**) The transmission spectrum of the NbTe_4_ nanosheets.

To evaluate the purity of the samples we collected, powder XRD analysis was performed using a Bruker D2 X-ray diffractometer equipped with Cu Kα radiation (λ = 1.5418 Å) at room temperature. The diffraction data were collected over a 2θ range of 10–70° with a step size of 0.02° and a fixed counting time of 1 s per step. As shown in Figure 4a, the XRD pattern of NbTe_4_ powder exhibits distinct diffraction peaks at (100), (002), (211), and (213) within the measured range. The experimental XRD pattern closely matches the calculated reference pattern, confirming the high purity of the synthesized NbTe_4_. As illustrated in Figure 4b, Raman spectroscopy shows characteristic peaks at 83 cm^−1^ and 129 cm^−1^, corresponding to Nb–C and Nb–O bonds, respectively, consistent with previous studies. Energy dispersive X-ray spectroscopy (EDS) mapping, as shown in Figure 4c, shows the characteristic peaks of Nb and Te, and their element ratio is close to 1:4, which is consistent with the stoichiometric ratio of NbTe_4_. This result further verifies the chemical purity and composition uniformity of the sample. The thickness of the NbTe_4_ nanosheets was determined using AFM, as depicted in Figure 4d,e. In agreement with previous reports, monolayer NbTe_4_ nanosheets exhibit an approximate thickness of 2.0 nm, which is consistent with the results of nanosheet thickness measured in Figure 4f. A Statistical analysis indicates that monolayers constitute approximately 54.9% of the total NbTe_4_ nanosheets. The morphology of the NbTe_4_ nanosheets was characterized using transmission electron microscopy (TEM), as depicted in Figure 4g,h. An HRTEM image, shown in Figure 4h, reveals the well-defined crystalline lattice structure (inset: corresponding diffraction image). UV-Vis-NIR spectroscopy was conducted on the NbTe_4_ dispersion. Figure 4i reveals a gradual decrease in absorption from 240 to 1200 nm. The absence of a distinct absorption peak suggests that NbTe_4_ exhibits broadband optical properties, similar to graphene.

### 3.2. Performance Characterization of Passively Q-Switched Lasers at 1.0 μm and 2.0 μm

**Figure 5:**
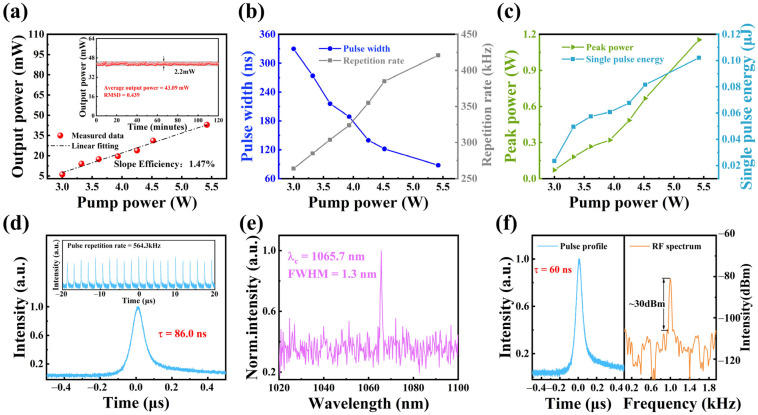
Characterization of PQS Nd:YAG laser. (**a**) Dependence of output power on pump power (inset: output power stability over two hours). (**b**) Pulse width and repetition rate as functions of pump power. (**c**) Dependence of peak power and single-pulse energy on pump power. (**d**) Minimum pulse duration (inset: corresponding stable pulse trains). (**e**) Emission spectrum of the PQS laser. (**f**) Minimum pulse duration and RF spectrum of the PQS laser under pulse-pumping operation.

For 1.0 μm laser operation, experimental results are shown in Figure 5. As seen in Figure 5a, the measured average output power exhibits a linear increase with pump power. The laser pump threshold is 3 W, and the maximum average output power reaches 44 mW at a pump power of 5.42 W. The inset depicts the stability of the PQS laser over a 2 h measurement period at a pump power of 5.42 W, with an average output power of 43.09 mW and a root-mean-square deviation (RMSD) of 0.439. The variation in pulse duration and repetition frequency with increasing pump power is illustrated in Figure 5b. As the pump power increases, the pulse duration decreases from 329 ns to 88 ns, while the repetition frequency rises from 263.9 kHz to 420.6 kHz. The shortest pulse width obtained in the experiment is 86 ns, with the corresponding pulse train shown in Figure 5d. Based on the above experimental data, the single pulse energy and peak power were calculated, as presented in Figure 5c. Both parameters exhibit a rising trend with increasing pump power: the peak power varies from 0.07 W to 1.15 W, while the single pulse energy ranges from 0.02 μJ to 0.1 μJ. The emission spectrum of the PQS laser at maximum output power is shown in Figure 5e, revealing a central wavelength (λ_c_) of 1065.7 nm and a full width at half maximum (FWHM) of 1.3 nm. Continuous pumping conditions can lead to an unstable PQS laser pulse output. To achieve a more stable laser pulse output and generate a pulse train at a defined frequency, pulse pumping was employed to optimize the output characteristics of the PQS laser. Figure 5f presents the radio frequency (RF) spectrum corresponding to the shortest pulse width of the PQS laser under pulse-pumping conditions. At a pulse-pumping frequency of 1 kHz and a pump power of 7.14 W, the output laser repetition frequency is 1 kHz, with an output power of 0.34 mW, a minimum pulse width of 60 ns, and a peak power of 5.66 W. The corresponding signal-to-noise ratio (SNR) is approximately 30 dBm. Notably, the pulse width obtained under pulse-pumping conditions is shorter than that observed under continuous pumping.

**Figure 6:**
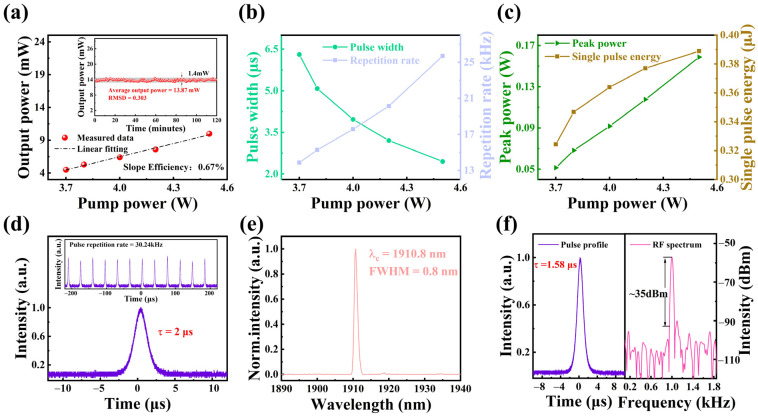
Characterization of PQS Tm:YLF laser. (**a**) Dependence of output power on pump power (inset: output power stability over two hours). (**b**) Pulse width and repetition rate as functions of pump power. (**c**) Dependence of peak power and single-pulse energy on pump power. (**d**) Minimum pulse duration (inset: corresponding stable pulse trains). (**e**) Emission spectrum of the PQS laser. (**f**) Minimum pulse duration and RF spectrum of the PQS laser under pulse-pumping operation.

To further verify the modulation performance of NbTe_4_ at 2.0 μm, the fabricated sample was incorporated into a Tm:YLF laser to achieve Q-switched operation. As illustrated in Figure 6a, the maximum average output power of the PQS laser is 13.87 mW, with a slope efficiency of 0.67%. The inset depicts the stability measurement of the maximum average output power of NbTe_4_ over two hours (range: 1.4 mW; RMSD: 0.303), demonstrating the excellent stability of NbTe_4_-SA in 2.0 μm PQS lasers. The relationship between pulse width, pulse repetition frequency, and pump power is shown in Figure 6b. As the pump power increases from 3.7 W to 4.5 W, the pulse width decreases from 6.3 μs to 2.45 μs, while the pulse repetition frequency increases from 13.8 kHz to 25.7 kHz. The shortest pulse width obtained in the experiment is 2 μs, with the corresponding pulse sequence illustrated in Figure 6d. Additionally, Figure 6c shows the trends of single pulse energy and peak power as a function of pump power. At a pump power of 4.5 W, the highest recorded pulse energy is 0.388 μJ, with a corresponding peak power of 0.16 W. Figure 6e shows the output spectrum of the PQS laser, indicating a central wavelength of 1910.8 nm and an FWHM of 0.8 nm. To achieve a more stable laser pulse output and generate a pulse train at a specific frequency, pulse pumping was used to optimize the output of the PQS laser. Figure 6f displays the minimum pulse width, the corresponding pulse train, the RF spectrum, and the associated SNR of the PQS laser under pulse-pumping conditions. At a pump power of 8.38 W and a pump pulse repetition frequency of 1 kHz, the output laser repetition frequency is 1 kHz, with an output power of 2.4 mW, a minimum pulse width of 1.58 μs, a peak power of 1.52 W, and an SNR of approximately 35 dBm.

### 3.3. Performance Characterization of Mode-Locked Lasers at 3.0 μm

**Figure 7:**
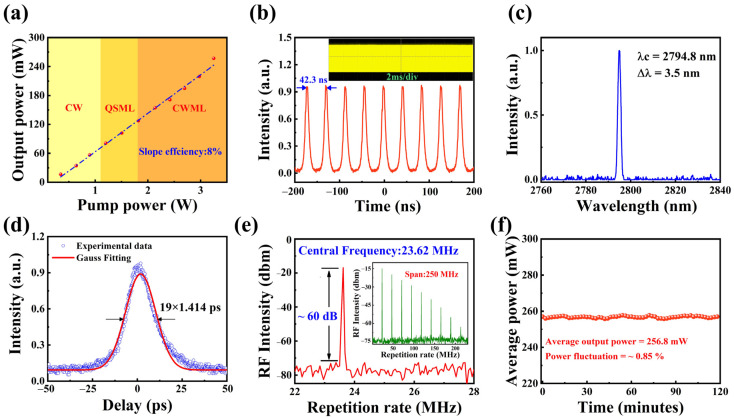
Characterization of the mode-locked Er-doped fluoride fiber laser. (**a**) Output power as a function of pump power. (**b**) Stable mode-locked pulse trains at different time scales. (**c**) Optical spectrum of the mode-locked pulses. (**d**) Autocorrelation trace with Gaussian fitting. (**e**) RF spectrum of the mode-locked pulses (inset: broadband RF spectrum). (**f**) Stability of the average output power over two hours.

The broadband saturable absorption properties of NbTe_4_-SA were investigated in the 3.0 μm spectral region. In an Er-doped fluoride fiber laser cavity, NbTe_4_-SA was deposited onto a gold mirror to enable mode-locked pulse generation. At a pump power of 0.3 W, the laser operated in the continuous-wave (CW) regime. When the incident pump power exceeded 1.2 W, the laser transitioned to Q-switched and subsequently to Q-switched mode-locked (QML) operation. Upon further increasing the pump power to 1.8 W, stable continuous-wave mode-locking (CWML) was achieved, yielding an average output power of 125 mW, as illustrated in Figure 7a. For pump powers ranging from 1.8 W to 3.5 W, the maximum output power reached 257 mW. The stable mode-locked operation demonstrated the high damage threshold of NbTe_4_-SA. A further increase in pump power led to instability in the mode-locked pulses. Figure 7b shows a typical stable mode-locked pulse train observed on both nanosecond and microsecond time scales. The pulses exhibit a well-defined and stable profile, indicating the effective modulation capability of NbTe_4_-SA. As illustrated in Figure 7c, the emission spectrum exhibits an FWHM of 3.5 nm, centered at 2794.8 nm. At the maximum average output power, an autocorrelator was used to measure the pulse profile. Under the assumption of a Gaussian pulse shape, the measured pulse duration was 19 ps, as shown in Figure 7d. The RF spectrum of the laser at the maximum output power is presented in Figure 7e. The observed repetition rate of 23.62 MHz corresponds to an effective cavity length of 4.8 m. The RF spectrum exhibits an SNR of approximately 60 dB, confirming stable mode-locked operation. Owing to the limited bandwidth of the detector (250 MHz), the amplitudes of higher-order harmonics attenuate rapidly, as shown in the inset of Figure 7e. The long-term stability of the maximum average output power was assessed over two hours, as depicted in Figure 7f. The recorded data indicate an average output power of 256.8 mW with a fluctuation of approximately 0.85%, demonstrating stable laser operation over two hours. Furthermore, no degradation or damage to the NbTe_4_-SA was observed throughout the experiment.

The experimental results show that NbTe_4_ exhibits different saturable absorption properties across specific wavelength bands (e.g., near-infrared and mid-infrared). These differences primarily arise from three factors: the material’s energy band structure, its nonlinear optical response, and the wavelength dependence of the absorption cross-section. When the incident photon energy (E = hν) is close to the material’s band gap (Eg) or corresponds to a specific energy level transition, the light–electron interaction is significantly enhanced. Moreover, saturable absorption is a nonlinear optical phenomenon governed by third-order nonlinear susceptibility (χ(3)). The intensity of this effect depends on the material’s nonlinear response and the spatial distribution of light intensity. This allows different spectral regions to induce distinct nonlinear interactions, such as two-photon absorption and free-carrier absorption. At the same time, the absorption cross-section will vary with wavelength, influencing the overall saturable absorption behavior. The interaction of various factors led to the different modulation capacities of NbTe_4_ from 1.0 μm to 3.0 μm.

Table 1 compares different PQS lasers utilizing broadband absorption SAs at 1.0 μm and 2.0 μm, as well as the PQS and mode-locked pulse parameters of these SAs at 3.0 μm. As shown in Table 1, the pulse width of the NbTe_4_-SA-based laser is 86 ns at 1.0 μm, 2 μs at 2.0 μm, and 19 ps at 3.0 μm. Notably, the pulse width of the 3.0 μm laser is less than half that of BP and graphene, while its peak power is comparable to BP and is higher than graphene. Furthermore, NbTe_4_ outperforms most conventional 2D materials at 1.0 μm and 3.0 μm. These findings demonstrate that NbTe_4_-SA-based lasers achieve narrower pulse widths, highlighting the exceptional pulse modulation capability of the material, particularly its ability to generate mode-locked pulses at 3.0 μm. Additionally, Q-switched and mode-locked operation remains achievable even after exposing NbTe_4_-SA to room temperature conditions for one month. In summary, NbTe_4_ is a promising SA device for lasers, exhibiting excellent broadband modulation capability and long-term stability.

## 4. Conclusions

In this study, NbTe_4_ powder was synthesized using a high-temperature solid-state method. Subsequently, high-performance NbTe_4_-SA was fabricated using the LPE method, and its optical parameters were characterized. The nonlinear optical characteristics of the synthesized SA, including broadband saturable absorption, were analyzed using a combination of Z-scan and P-scan techniques. The measured saturation intensities were 59.53 GW/cm^2^ at 1.0 μm with a modulation depth of 17.4%. It is 14 GW/cm^2^ and 5.3% at 2.0 μm and 6.8 MW/cm^2^ and 21.5% at 3.0 μm. To the best of our knowledge, this study represents the first demonstration of NbTe_4_-SA-based lasers operating in the 1.0, 2.0, and 3.0 μm bands. PQS was successfully achieved at 1.0 μm and 2.0 μm, yielding pulse durations of 86 ns and 2 μs, respectively. A mode-locked pulse with a duration of 19 ps was generated at 3.0 μm, confirming picosecond operation. These findings highlight the potential of NbTe_4_ for nonlinear optical modulation and pave the way for the development of other quasi-1D broadband saturable absorber materials.

## Figures and Tables

**Table 1:** Performance comparison of lasers based on different broadband saturable absorbers.

SA	λ (μm)	Operation Mode	Pulse Width (ns)	Output Power (mW)	Repetition Rate (kHz)	Single Pulse Energy (μJ)	Peak Power (W)	Ref.
BP	1.0	PQS	495	22	312	0.07	0.14	[2]
	2.0	PQS	1780	151	19.25	7.84	4.4	[30]
	3.0	CWML	0.042	613	24,270	0.0255	607	[31]
MoS_2_	1.0	PQS	970	227	732	0.31	0.32	[32]
	2.0	PQS	800	100	48.09	2.08	2.6	[33]
	3.0	PQS	806	140	70	2	2.48	[34]
VTe_2_	1.0	PQS	195	88	350	0.25	1.29	[35]
	2.0	PQS	563	237	62.5	3.79	6.73	
	3.0	PQS	749	402.8	129.8	3.1	4.14	
Graphene	1.0	PQS	753	266	436	0.61	0.81	[36]
	2.0	PQS	2080	38	18.1	1.74	0.84	[37]
	3.0	CWML	0.042	18	25,400	0.0007	16.87	[1]
Bi_2_Te_3_	1.0	PQS	2000	183	151.5	1.21	0.6	[38]
	2.0	PQS	382	272	57.67	4.8	12	[39]
	3.0	CWML	0.021	360	42,430	0.008	400	[40]
Ta_2_NiTe_5_	1.0	PQS	330	273	192	1.42	4.36	[41]
	2.0	PQS	2600	780	22	35.1	13.5	
	3.0	PQS	850	182	125	1.46	1.71	
NbTe_4_	1.0	PQS	86	43.09	420.6	0.1	1.15	This work
	2.0	PQS	2000	13.87	25.7	0.388	0.16	
	3.0	CWML	0.019	257	23,620	0.011	570	

**Figure A1:**
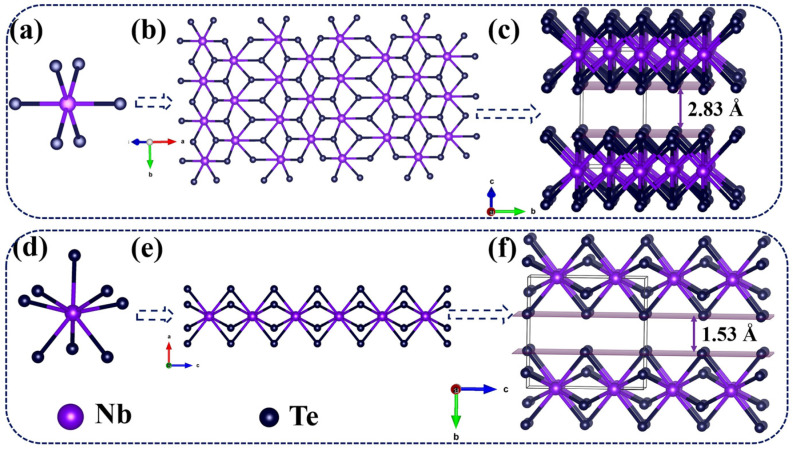
Crystal structures of NbTe_2_ and NbTe_4_. (**a**) NbTe₆ coordination in NbTe_2_; (**b**) two-dimensional layered structure of NbTe_2_; (**c**) multilayer stacking in NbTe_2_ with an interlayer spacing of 2.83 Å; (**d**) NbTe₈ coordination in NbTe_4_; (**e**) one-dimensional chain structure of NbTe_4_; (**f**) multichain stacking in NbTe_4_ with an interchain spacing of 1.53 Å.

**Figure A2:**
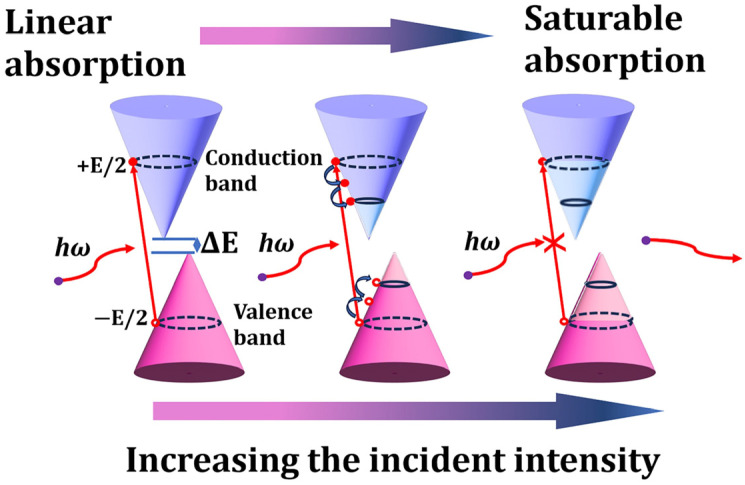
Schematic illustration of the saturable absorption mechanism in NbTe_4_.

## Data Availability

The results presented in this paper are not publicly available but can be obtained from the authors upon reasonable request.

## Abbreviations

The following abbreviations are used in this manuscript:

quasi-1D	Quasi one dimensional
2D	Two dimensional
TMCs	Transition metal chalcogenides
SA	Saturable absorber
PQS	Passively Q-switched
NLO	Nonlinear optical
vdW	van der Waals
CDWs	Charge density waves
SC	Superconductivity
SWCNTs	Single-walled carbon nanotubes
TMTCs	Transition metal trichalcogenides
LPE	Liquid-phase exfoliation
XRD	X-ray diffraction
EDS	Energy dispersive X-ray spectroscopy
AFM	Atomic force microscopy
TEM	Transmission electron microscopy
HRTEM	High-resolution transmission electron microscopy
IPA	Isopropyl alcohol
OA	Open aperture
OC	Output coupler
NA	Numerical aperture
RMSD	Root-mean-square deviation
FWHM	Full width at half maximum
RF	Radio frequency
SNR	Signal-to-noise ratio
CW	Continuous wave
QML	Q-switched mode-locked
CWML	Continuous-wave mode-locking

## Appendix A

### Appendix A.1

The van der Waals interactions between adjacent chains of a 2D material (e.g., NbTe_2_) compared to a quasi-1D material (e.g., NbTe_4_) (Figure A1).

### Appendix A.2

Due to variations in interlayer coupling strength and the effects of quantum confinement, exfoliated NbTe_4_ nanosheets typically exhibit semiconducting characteristics. The saturable absorption mechanism of NbTe_4_ can be understood based on the Pauli blocking principle, as illustrated in Figure A2. When the incident light intensity exceeds the bandgap energy (ΔE), it can excite electrons from the valence band (pink) to the conduction band (blue). Within a few hundred femtoseconds, these excited carriers undergo rapid thermalization, forming a hot Fermi–Dirac distribution. As a result, the generation of electron–hole pairs suppresses further interband photon transitions at energy level E. Subsequently, due to intraband phonon scattering, the carriers undergo cooling, establishing a new equilibrium between electron–hole pairs and enabling linear optical transitions at low light intensities. Under high-intensity illumination, the carriers excited by photons quickly occupy energy states near the conduction and valence band edges. This leads to additional absorption saturation, a phenomenon governed by the Pauli blocking principle.

## Appendix B

### Appendix B.1. P-Scan Experimental Setup

The P-scan experiment was conducted using a homemade 2790 nm SESAM-based passively mode-locked fiber laser, which has a pulse duration of 10 ps and a repetition frequency of 23.62 MHz. A 50:50 beam splitter, positioned at a 45° angle, was used to divide the laser beam into two paths. The reflected beam was directed to a power meter (Dynamometer 1) to serve as a reference. Meanwhile, the transmitted beam was focused onto the surface of the NbTe_4_-coated gold mirror, which was slightly tilted to reflect the beam toward a second power meter (Dynamometer 2). By varying the laser output power, a series of reflectance measurements were obtained.

### Appendix B.2. 2.0 μm Laser Experimental Setup

For the 2.0 μm laser experiment, a laser system incorporating a Tm:YLF crystal (3 × 3 × 10 mm^3^; 3.0 at.%; A-cut) as the gain medium was designed. The experimental setup is illustrated in Figure 3a. A commercial 793 nm fiber-coupled diode laser (fiber core diameter: 200 μm; NA: 0.22) served as the pump source, providing a maximum output power of 20 W. A 1:0.8 collimation focusing system was employed to precisely focus the pump beam onto the crystal with a spot diameter of approximately 160 μm. The crystal was coated at both ends with different dielectric coatings: the input facet (M1) was coated for high transmittance at 793 nm (HT@793 nm) and high reflectance at 1908 nm (HR@1908 nm), while the output facet had an anti-reflective coating at 1908 nm (AR@1908 nm). The output coupler was a concave mirror with a radius of curvature of 50 mm, featuring 10% transmittance in the 1900–2100 nm range. The total resonator length was 25 mm.

### Appendix B.3. Measurement Instruments for 1.0 μm and 2.0 μm PQS Laser Characterization

The experimental setups for PQS lasers based on NbTe_4_-SA at 1.0 μm and 2.0 μm are depicted in Figure 3a. The 1.0 μm laser output was measured using a power meter (30A-P-17, Ophir Optronics Solutions Ltd., Jerusalem, Israel), while the 2.0 μm laser output was recorded using a different power meter (LPE-1601, Physcience Opto-Electronics Ltd., Beijing, China). Pulse train signals for the 1.0 μm and 2.0 μm PQS lasers were detected using InAsSb and InGaAs photodetectors (DET10A/M and PDA10D2, Thorlabs, Inc., Newton, NJ, USA), respectively. A high-speed oscilloscope (DPO4104B, Tektronix, Inc., Beaverton, OR, USA) with a 1 GHz bandwidth and a 5 GHz sampling rate was employed to capture the pulse train signals. The spectral characteristics of the lasers were measured using two spectrometers: an Ocean Optics USB4000-VIS-NIR for the 1.0 μm wavelength range and a Wavescan MIR (Angewandte Physik & Elektronik GmbH, Berlin, Germany) for the 2.0 μm range.

### Appendix B.4. Measurement Setup for 3.0 μm Mode-Locked Fiber Laser Characterization

For the characterization of the 3.0 μm fiber laser, the output pulse was measured through a germanium broadband filter (WG91050-C9, Thorlabs), which exhibited a measured transmittance of approximately 89% at 2780 nm. The output power was recorded using a power meter (3A-P, Ophir Optronics Solutions Ltd., Jerusalem, Israel). The mode-locked pulse train was detected using a HgCdTe photoconductive detector (PCI-9, VIGO System, Warsaw, Poland) with a 250 MHz bandwidth and was recorded using an oscilloscope (MDO3104, Tektronix Inc., Beaverton, OR, USA) with a 1 GHz bandwidth. The corresponding mode-locked spectrum was obtained using an optical spectrum analyzer (Ocean Optics SIR 5000) with a resolution of 2 nm.
